# Production of Cost-Effective Mesoporous Materials from Prawn Shell Hydrocarbonization

**DOI:** 10.1186/s11671-016-1634-z

**Published:** 2016-09-29

**Authors:** S. Román, B. Ledesma, A. Álvarez-Murillo, E. Sabio, J. F. González, C. M. González

**Affiliations:** Departamento de Física Aplicada, Escuela de Ingenierías Industriales, Universidad de Extremadura, Avda. Elvas, s/n, 06006 Badajoz, Spain

**Keywords:** Hydrocarbonization, Mesoporous materials, Prawn shells, p-Nitrophenol

## Abstract

In this work, prawn shell was studied as raw material for the production of mesoporous adsorbents via hydrocarbonization, studying the effect of temperature and time on the process reactivity and final characteristics of the hydrochars. By suitable characterization technique analyses (N_2_ adsorption at 77 K, SEM observation, ultimate analysis, surface composition), the materials were examined. It was found that in both cases mesoporous materials with low values of *S*_BET_ due to the presence of CaCO_3_ on the material structure. In order to provide a potential application for these materials, the adsorption behaviour of hydrochars (HCs) as well as that of pristine prawn shells and ashes from prawn shell combustion was studied for the first time with the model compound p-nitrophenol (PNP). The results indicated that HC treatment was beneficial and enhanced adsorption performance, especially at high values of equilibrium concentration, attaining adsorption capacities up to 1.6 mg g^−1^.

**Electronic supplementary material:**

The online version of this article (doi:10.1186/s11671-016-1634-z) contains supplementary material, which is available to authorized users.

## Background

The search on new precursors for adsorbent manufacturing is a threat for researchers worldwide, owed to the great increase on activated carbon (AC) demand during the last decades. In fact, in the frame of challenge “Climate Action, Environment, Resource Efficiency and Raw Materials”, within the Horizon 2020 Strategic Programming, the strategic area “Waste: a resource to recycle and reuse and recover raw materials”, claims for a sustainable recycling of wastes. In this sense, the main concern is to support the transition towards a more circular economy, where the waste generated in one industry becomes a secondary raw material for another industry (which is called “industrial symbiosis”). The strategy widens the spectrum of wastes to food, agroindustrial, buildings, etc. [1]. This transition will generate growth and jobs while contributing to environmental protection and reducing Europe’s dependency on raw material imports.

The abovementioned reasons have motivated scientifics all over the world to investigate novel precursors for preparing added-value materials and, in particular, carbon materials. Classical lignocellulosic materials (fruit stones and shells, wood, cork…) have widely proven their suitability to yield biofuels with high heating value, by previous carbonization, provided their high carbon content. Frequently, these raw materials can also be regarded as a source to produce activated carbons (ACs) via chemical of physical activation [2].

There are, however, other types of wastes, which are discarded for recycling via the former processes, because of their high proportion of inorganic material; this is the case of crustacean wastes. Prawn shells, for example, are an abundant and non-centralized waste annually produced and discarded.

Besides the search for new precursors, the strategy also claims for the need to improve production processes in order to make them more energetically favourable, and also lower carbon dioxide emissions. Recently, hydrocarbonization (HTC) has proven to be a promising step in the production of cost effective carbon materials. By HTC, biomass was subjected to mild temperature conditions (150–350 °C) in a closed aqueous system. Under these conditions, the material starts undergoing hydrolysis reactions (the kinetics is strongly dependent on the heating conditions) which occur in parallel or in series with many other processes (decarboxylation, dehydration, polymerization…) [3]. Moreover, as hydrolysis occurs, more hydronium ions are produced which further enhances decomposition reactions. As a result, a wide variety of chemical compounds migrate to the liquid phase, and some gases are also formed, which causes an increase in the system pressure. After a period of time, a solid product called hydrochar (HC) is obtained. Several advantages associated to this process make it appealing. Firstly, it is a very simple, cost-effective process, as compared to other traditional thermochemical processes such as pyrolysis or gasification. In opposition to these processes, HTC temperatures are moderate and no gas feeding is needed. Thermodynamically, it is a very favourable process, since most of the occurring reactions are exothermic and the hydronium ions generated as a consequence of primary decomposition, act as catalysts of the process. Autogenous pressure is also a reaction promotor factor; it is produced as a result of gasification reactions in the bulk of the liquid phase, with no gases leaving the reactor, which is tightly closed; this also results in a null emission of harmful gaseous species. Finally, it is also worth mentioning that previous pieces of research have reported other benefits related to the thermal processing of the solid phase (HC) such as ease to pelletize [4], decrease of boiling ash temperature, or improvement of emissions during gasification [5], as compared to the initial precursor.

On the other hand, HCs have an incipient porosity, which can make them suitable to be used as adsorbents. Also, their particular surface chemistry can be tuned up to favour selectivity towards a given adsorbate [6].

Crustacean market has experimented a continuous increase during the last years. According to recent reports [7], the world annual consumption of general crustacean species and of prawn shell, are, respectively, above 6  ·10^6^ and 6  ·10^4^ ton. The convenience of searching uses for these materials has preferently focused on the extraction of chitin and chitosan [8]. By acid washing, CaCO_3_ is easily dissolved, yielding chitin, and subsequent treatment with alkaline solution helps the extraction of proteins to yield chitosan. Both materials have found a field of use on biomedical and pharmaceutical applications such as drug release, wound dressing or biofilms.

The potential preparation of carbon materials by thermochemical processing from these wastes has been addressed recently; among the pieces of research on this topic, the work of White et al. is outstanding [9]; these researchers achieved the preparation of N-rich carbon materials by the joint of HTC followed by acid washing with acetic acid and pyrolysis, and obtained mesoporous activated carbons (ACs) with high values of specific surface area. Gao et al. [10] followed a similar recipe, but they added an additional alkaline washing step in order to extract proteins from the material; then, they studied the potential use of the ACs as electrode material for supercapacitators. In this line, Slivak et al. [11] also combined acid and alkaline washing with CO_2_ activation, which improved the porous development and enhanced charge propagation.

Regarding adsorption processes of prawn shell carbon materials, the bibliography only provides works dealing with the use of ACs obtained by chemical activation prepared by traditional methods. For example, Arulkumar et al. [12] and Qin et al. [13] studied the performance of adsorbents derived from prawn shell chemical activation on chromium removal and antibiotics, respectively.

Up to date, the production and application of HCs as adsorbents, to the best of the authors known, has not been studied before.

With these antecedents, the present work focused on the following objectives:

- Preparation and characterization of prawn-shell derived HCs, studying the effect of experimental variables on the reactivity, final porosity and chemical properties of the obtained materials
- Application of the prepared HCs as adsorbents for a major waste water contaminant, p-nitrophenol, and suggestion of the corresponding adsorption mechanisms

## Methods

### Raw Materials

Prawn shells were provided by a shellfish industry (La Mar) located in Extremadura Region (Southwest Spain). The industry, which sells both shelled and pristine prawn shells, collected the shells by manual shelling and frozen them for further use. Their transportation was made in closed containers and, once received, the residues were dried overnight at 80 °C and ground to a particle size of 0.5–1.0 mm diameter. After drying, they were stored in closed flasks placed in a desiccator for further use.

### Preparation of Hydrochars

The HTC processes were performed in a stainless steel autoclave (Parr, USA). In a 1.2L teflon vessel (unstirred), 60 g of dried prawn shell and 0.9 L of deionized water at room temperature were added; the system was sealed and placed into the autoclave and the system remained overnight at room temperature. After this, the system was heated up in an electric furnace at selected temperatures (180, 200, 220 °C), during a chosen processing time (2.5, 5, 10, 15 and 20 h). When the reaction time was reached, the autoclave was removed from the oven and subsequently placed in a cold-water bath. After cooling, the solid phase was separated from liquid by vacuum filtration and subsequently dried at 80 °C to remove residual moisture. The dried HCs were stored in closed flasks placed into a desiccator until further analysis.

### Characterization Techniques

The pristine raw material was firstly characterized in terms of their proximate and ultimate analyses, following the corresponding technical specifications [14], using a muffle oven (Hobersal model 12PR330CCH). Besides, following the procedure of the former norm, prawn shell ashes were obtained and separated for further use.

Elemental analyses (for C, H, N, O) on both the raw material and derived HCs were carried out with an elementary analyser (Eurovector EA 3000), according to the norm CEN/TS 15104 (for determining the content of C, H and N) and CEN/TS 15289 (for S) (CEN/TS 335 Biomass standards).

The porosity of the materials was examined by N_2_ adsorption at 77 K (AUTOSORB 1, Quantachrome); before analyses the samples were outgassed overnight at 80 °C. Finally the surface features of the HCs was examined by SEM imaging with a Hitachi S-3600N Microscope; SEM samples were prepared by depositing 50 mg of sample individually on Al studs, covered with conductive adhesive carbon tapes and then coating with Rd-Pd for 1 min to prevent charging during observations. Imaging was done in the high vacuum mode at an accelerating voltage of 20 kV, using secondary electrons.

### Adsorption Studies

PNP equilibrium adsorption isotherms were determined on the basis of batch analysis. For this purpose, a fixed amount of 0.1 g of adsorbent was added to 15 mL of organic solution (adsorbate with ultrapure water at neutral non-fixed pH) with initial concentrations ranging 10–100 mg L^−1^. The flasks were then placed in a thermostatic bath at 298 K and allowed to equilibrate for 48 h, since previous experimentation on the adsorption kinetics showed that this period of time was enough to guarantee equilibrium.

After equilibration, the adsorbents were filtered and the concentration of PNP in the supernatant solutions was analysed by UV/vis spectrophotometry (spectrophotometer UNICAM Helios-λ) at a wavelength of 225 nm. This wavelength was selected after previous spectral scanning tests, which showed the stability of this signal (λmax), independently of the possible pH variations. Then, the absorbances of a series of standard solutions of different concentrations were measured and the suitability of Beer’s law was confirmed. The regression coefficients were very close to one under the range of concentrations used in this study.

## Results and Discussion

### Raw Material

Table 1 shows the immediate and proximate analysis of prawn shell. At first glance, it is worthy to mention the high inorganic content of the material, as revealed by the ash proportion, which involves a drastic difference in comparison with other biomass materials.

**Table 1:** Immediate and proximate analysis of prawn shell (%)

Fixed carbon	Volatile matter	Ashes	Moisture	N, %	C, %	H, %	O, %
7.32	65.16 (61.3)	23.11	4.4 (6)	8.82	34.95	5.23	51.0

The low organic content of this material implies the discard of this material as biofuel and strengths the interest of searching other uses for it. Regarding its potential use as biosorbent, the presence of carbon on the material can be beneficial, since it can help the prominence of certain dispersive adsorption interactions, as it will be described further in the “Surface Characterization of Hydrochars” section; at this point, it is interesting to remark that, although low, other biominerals such as sea shells have even a lower organic content, which would justify the choice of prawn shells for potential adsorption applications, especially if aromatic compounds are considered [15].

On the other hand, the presence of heteroatoms on the precursor can also be of interest for several applications. Many researchers have tried to dope carbon materials with N, due to the benefits it entails regarding their conductive behaviour, for example [10, 11]. In the frame of wastewater treatment, specific adsorption processes are favoured for carbons with N-rich surface functionalities, as it is the case, for example, of CH_3_I isotopes [16] or phenol compounds [17]. Oxygen proportion is also outstanding in this material, which is probably combined in a great extent as mineral carbonates, such as CaCO_3_ and MgCO_3_.

The thermal behaviour of prawn shell was studied by means of thermogravimetric analysis under inert atmosphere. The TG/DTG obtained, not shown here for the sake of brevity (provided as Additional file 1: Figure S1), revealed that the decomposition of this material occurs along different stages. The most important weight loss is produced at 200 °C approximately, followed by two consecutive decomposition stages. Later on, a new drop of weight is found around 700 °C. At 750 °C, 63 % of the initial mass has been decomposed; the slight difference between this value and the volatile matter proportion shown in Table 1 (65.2 %) is consistent with the lower final temperature employed in TG analysis.

### Hidrocarbonization Experiments

**Fig. 1:**
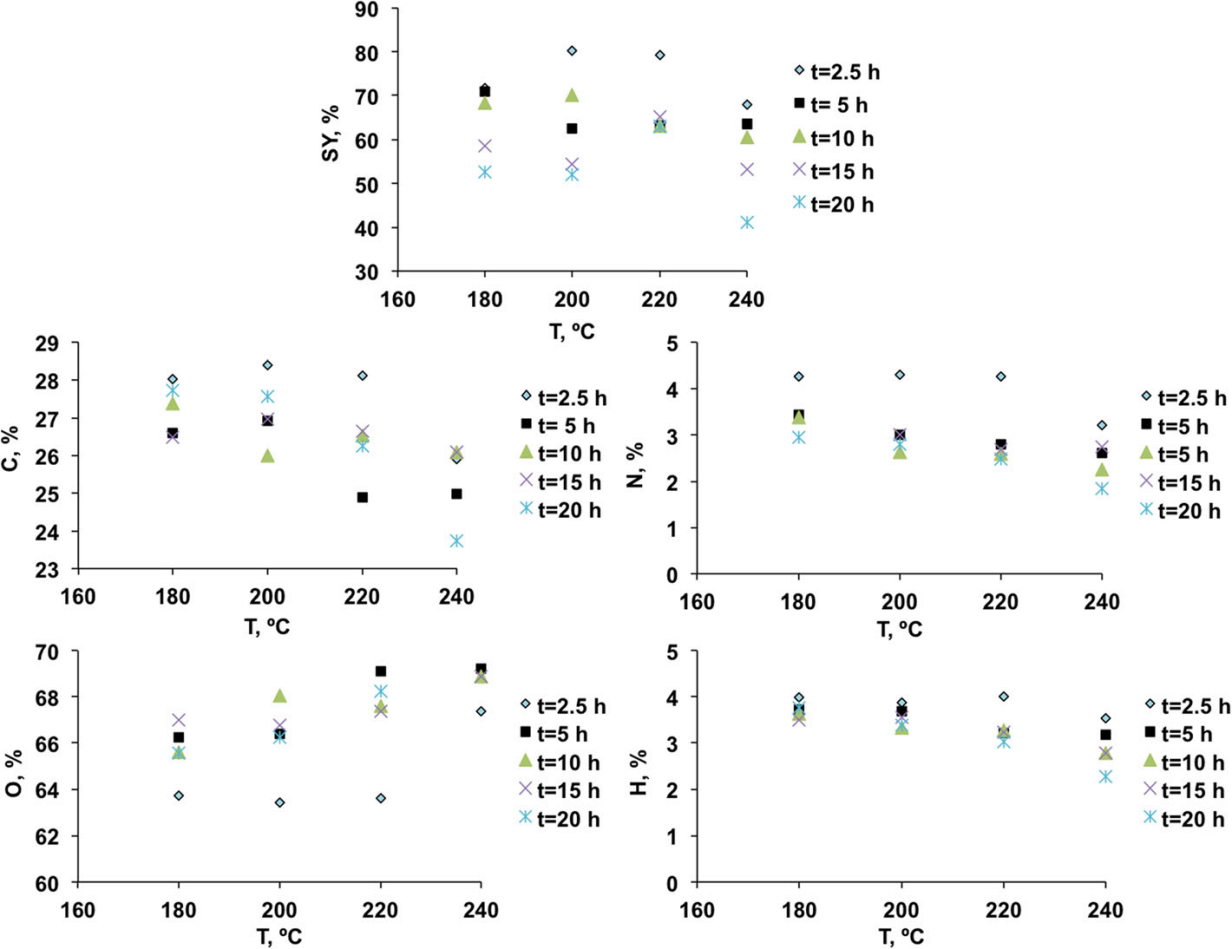
Evolution of solid yield (SY) and elemental analysis with HTC conditions

Table 2 collects the experimental conditions of the different HTC runs and the corresponding solid yields and elemental composition of the prepared HCs. Each experiment was named according to the nomenclature PS/*T*/*t*, where *T* stands for temperature and *t* represents the time period. In order to favour the interpretation of tendencies, some graphs have been plotted and are included in Fig. 1. Several remarks can be made from these results.

**Table 2:** Solid yield values and elemental composition of HCs

	SY, %	N, %	C, %	H, %	O, %
PS/180/2.5	71.6	4.27	28.03	3.98	63.72
PS/180/5	70.8	3.43	26.61	3.73	66.23
PS/180/10	68.4	3.38	27.39	3.62	65.62
PS/180/15	58.5	3.01	26.49	3.49	67.01
PS/180/20	52.6	2.95	27.74	3.76	65.55
PS/200/2.5	80.3	4.31	28.4	3.87	63.42
PS/200/5	62.5	3.00	26.93	3.69	66.38
PS/200/10	70.0	2.63	25.99	3.33	68.05
PS/200/15	54.5	2.69	26.97	3.56	66.78
PS/200/20	52.0	2.79	27.56	3.39	66.26
PS/220/2.5	79.3	4.26	28.12	4.02	63.62
PS/220/5	63.2	2.80	24.91	3.21	69.09
PS/220/10	63.1	2.59	26.53	3.27	67.61
PS/220/15	65.1	2.74	26.65	3.24	67.37
PS/220/20	63.0	2.48	26.26	3.03	68.23
PS/240/2.5	67.9	3.21	25.93	3.54	67.35
PS/240/5	63.5	2.61	24.99	3.18	69.22
PS/240/10	60.5	2.25	26.09	2.79	68.87
PS/240/15	53.2	2.25	26.09	2.79	68.87
PS/240/20	41.2	1.85	23.74	2.27	72.14

#### Solid Yield

In the first place, an interesting point is that in general the values of solid yield are greater than those found under similar experimental conditions for other biomass residues. This fact, directly related to the lower volatile content of prawn shells as compared to those residues, can be seen as a significant strength to face a large-scale production.

Regarding the influence of experimental variables, the effect of temperature will be analysed first. Most of the research made on HTC agrees on the fact that a higher temperature involves a decrease on the solid yield as a result of the enhancement of primary decomposition reactions. In this work, this effect is dependent on the dwell time considered and is more marked when longer periods of time are examined. The effect found for low periods of time (2.5 h series) is surprising for the lower temperature range (180–200 °C). In this period, it seems that 200 °C involves a greater solid yield than 180 °C.

On the other hand, the effect of HTC time is in general positive, especially in the case of higher temperatures. It seems that this variable has a greater influence on the solid yield than the temperature. This finding is a highlight and is not frequent for other materials subjected to HTC under similar conditions. To the best of the authors’ knowledge, there are no previous pieces of research on the effect of HTC time on crustacean-derived materials. Previous pieces of research devoted to the study of chitin extraction give evidence about the little knowledge on the factors influencing its solubility [8].

#### Elemental Composition

The distribution of elements between the solid and liquid phase is very interesting not only because it helps in understanding the occurrence of decomposition reactions but also because the permanence of certain elements on the HC can provide particular applications for these materials. However, the identification of the decomposition stages is a very complex task, because, during HTC, many different equilibria (hydrolysis of the extractives, dehydration, decarboxylation, condensation, polymerization, aromatization in the liquid phase…) take place in parallel or in series, depending on the case [18].

In the first place, clear tendencies are observed in the case of O and H. While the first one increases as the reaction severity increases (i.e., greater temperature or time), the second one decreases. Secondly, C follows the tendencies identified previously for solid yield values, which is consistent with the primary decomposition of the polysaccharide components of the prawn shells.

With respect to N, little is known about its fate during HTC processes, and it depends on the type of molecule from which it is degraded. Coronella et al. [19] studied the distribution of elements on the liquid and solid phases obtained from HTC of cow manure and found that for low time periods (even as low as 5 min) N migrated to the liquid phase; then, N was again absorbed onto the carbon surface, and this effect was more marked for greater time periods and temperature.

### Surface Characterization of Hydrochars

**Fig. 2:**
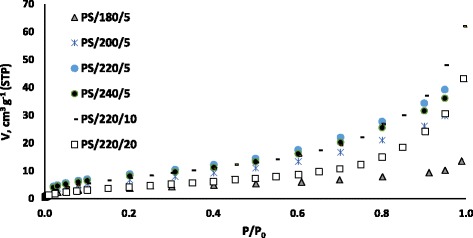
Adsorption isotherm of selected HCs

The porous structure of the HCs was studied, firstly, by N_2_ adsorption adsorption at 77 K. It was found, that in all cases, very similar results were found, independently of the reaction conditions. In all cases, clear mesoporous materials were obtained, with low values of apparent surface, ranging between 15 and 40 m^2^ g^−1^. As an example, Fig. 2 shows the adsorption isotherms of some selected samples which were chosen because they have differences on their solid yield values, and therefore, their comparison might offer some insight about the reaction effects.

As inferred from the isotherms, N_2_ volume is poor for low values of relative pressure, and it grows gradually as pressure is increased. This behaviour is typical of type IV isotherms, according to BBDT classification and is indicative of mesoporous materials [20]. No significant differences on the shape of the isotherms were identified. Only a slight decrease of the adsorption volume was observed in the case of the sample prepared at the lowest temperature (PS/180/5).

Other authors have also reported the predominance of mesopores on prawn-shell derived carbon materials. For example, White et al. [9] studied the structure of carbon materials obtained from prawn shells by HTC at 220 °C followed by pyrolysis at 750 °C and found that mesoporous materials with large pores were obtained. They related these results to the permanence of CaCO_3_ on the polysaccharide structure of the precursor and proposed a way of removing this mineral by washing the material with acetic acid. After this treatment, the accessible pore volume increased significantly (*S*_BET_ around 300 m^2^ g^−1^), and the mean mesopore diameter decreased as a result of microporosity un-blockage and widening.

Other authors have used other acids to remove the CaCO_3_ from prawn shells, such as HCl [10] or H_2_SO_4_ [13]. A subsequent treatment with a base such as NaOH and KOH was applied to eliminate the chitin and chitosan, respectively [13].

**Fig. 3:**
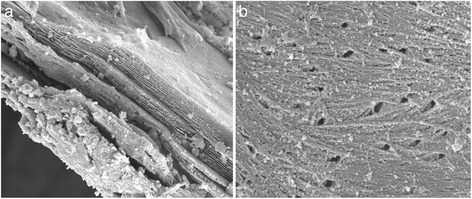
SEM images of PS/220/5 under different magnifications: **a** 5500 and **b** 20,000

**Fig. 4:**
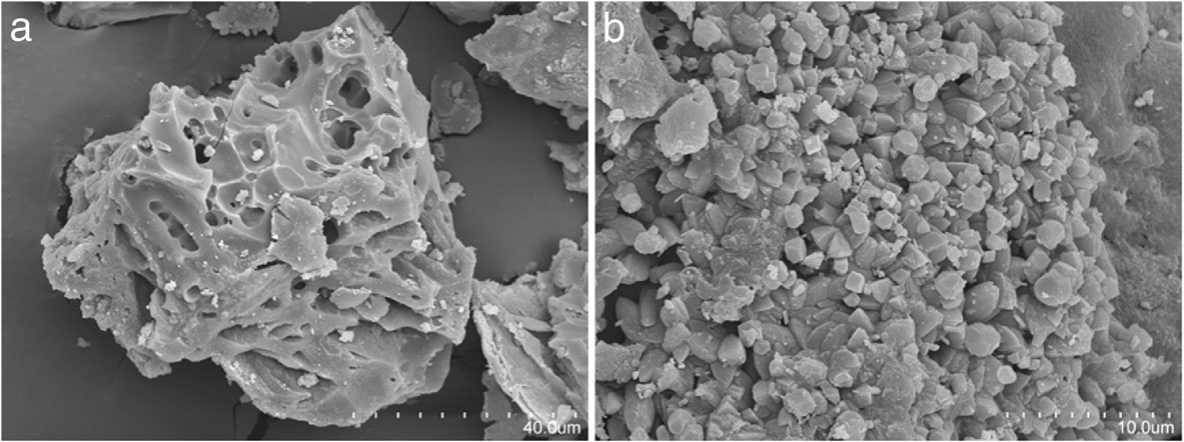
SEM images of PS/220/20 under different magnifications: **a** 250 and **b** 1500

The surface morphology of the HCs was studied by SEM examination. In Figs. 3 and 4, some of the micrographies obtained for selected HCs (PS/220/5 and PS/220/20) have been collected, under different magnifications.

In all cases, the images reveal the existence of large cavities and tunnels on the HCs surface, and more or less cylindrical pores. Moreover, some particles are found on the materials, which can be attributed to the inherent mineral content of the HCs. In situ EDX analyses confirmed the mineral character of these particles, with high quantities of Ca, K, Mg and others (see Table 3). Arulkumar et al. [13] found these particles on the surface of ACs obtained by chemical activation of prawn shells.

**Table 3:** Surface composition of HCs by EDX

	O	C	Ca	P	Mg	S	Na
PS/220/5	39.1	42.5	12.2	3.5	1.9	0.62	ND
PS/220/20	56.9	22.9	16.1	2.7	0.68	0.37	0.22

The results collected in Table 3 showed that increasing the HC time was consistent with lower C surface content, as a result of primary decomposition reactions. As a consequence, O and Ca fractions clearly increased; the other elements did not show remarkable differences.

### Adsorption Study

The adsorption of p-nitrophenol was studied on three different systems: pristine prawn shell, hydrocarbonized prawn shell (HC/220/2.5) and, provided the high ash content of the raw material, an additional study onto the ash derived from prawn shell combustion was also studied.

Sample HC/220/2.5 was selected among all the HC samples produced because, as compared to the rest of the HCs, it had advantageous chemistry surface due to its high C and N content, which can favour both specific and non-specific interactions with the adsorbate, as it is explained below.

**Fig. 5:**
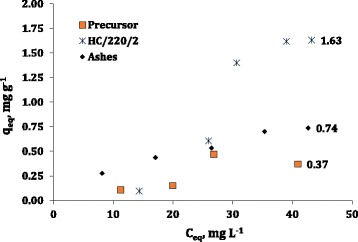
p-Nitrophenol adsorption isotherms

In Fig. 5, the corresponding equilibrium isotherms have been collected. From the plot, it is evident that the adsorption of this adsorbate is not favoured at low values of equilibrium concentration. The values of *q*_0_ only increase if a certain amount of p-nitrophenol is present on the bulk of the liquid phase. According to the classification proposed by Giles et al. [21], this shape with low adsorption affinity at low *C*_e_ values, corresponds to L-type isotherms. This type, very common for aromatic species, is indicative of a flat horizontal configuration of the adsorbate on the adsorbent surface and of low competition between the adsorbate and the solvent to occupy the adsorption site.

A more detailed examination reveals that there are remarkable differences between ashes and the other two materials; while in the first case there is a gradual increase of the adsorbent amount, the other two adsorbates exhibit a two-step adsorption behaviour. Previous studies on the adsorption of phenol compounds have found this two-step feature and have related it to a change in the orientation under which the molecule is adsorbed (from horizontal to vertical) [22].

The results obtained clearly show that subjecting prawn shells to hydrocarbonization is beneficial for subsequent adsorption of PNP; this treatment involved a 4-fold increase of the adsorption capacity of the pristine material.

The differences on the behaviour of the three materials can be explained on the basis of both their porous structure their surface chemistry. The first factor is advantageous for the HC, in comparison to the other two materials. Regarding the second factor, most of the research made on the adsorption of organic molecules agrees on the fact that both specific and non-specific interactions have to be considered. In the case of specific interactions, the N at the carbon surface might form hydrogen bonds with the OH substituent [23]. With respect to non-specific or dispersive interactions, the HC turbostratic structure formed as a result of the precursor thermal treatment will favour adsorption by non-specific П–П interactions between the delocalized electrons found on the edges of the carbon layers and the benzene rings of the adsorbate [24].

## Conclusions

In this work, prawn shells were subjected to hydrocarbonization processes under different experimental conditions, studying their effect on the process reactivity and elemental composition of HCs. It was found that, in general, solid yield was lower for longer treatments, while the effect of temperature was not so clear, indicating that this parameter affects both degradation and recombination reactions.

The elemental composition of the produced HCs also changed as a result of experimental conditions, which suggested some reaction pathways, such as dehydration, N migration and subsequent absorption or polysaccharide decomposition.

Regarding their porosity, all HCs were very mesoporous and had low values of *S*_BET_, as a result of the permanence of CaCO_3_ on the materials; however, despite this low porosity development, they exhibited a joint of surface properties which combined both available aromaticity and specific surface groups including a moderate quantity of N (above 4 % *w*/*w*) in some cases.

p-Nitrophenol adsorption was tested on the pristine material, on the derived-ashes and on one selected hydrochar (sample PS/2.5/220). It was found that the HC provided the greatest removal, followed by ashes. The reasons beyond these results were examined and the adsorption mechanism was proposed as a combination of specific interactions (as hydrogen bond) and non-specific ones (including dispersive interactions between the aromatic PNP ring and the delocalized electrons of the HC basal planes).

Future research will study the modification of both structural and surface chemistry properties of the HCs in order to broaden the spectrum of applications, such as porous supports for heterogeneous catalysis or energy storage device production.

## Additional file

Additional file 1: Figure S1. TG/DTG profiles of pristine prawn shell. (DOCX 31 kb)
