# Supplementary material for: Production of Cost-Effective Mesoporous Materials from Prawn Shell Hydrocarbonization
Source: Nanoscale Res Lett. 2016 Sep 29;11:435. doi: 10.1186/s11671-016-1634-z (PMC5040653; doi:10.1186/s11671-016-1634-z)
Supplement: Additional file 1: Figure S1. — TG/DTG profiles of pristine prawn shell. (DOCX 31 kb) [file 11671_2016_1634_MOESM1_ESM.docx]

**Figure S1.** TG/DTG profiles of pristine prawn shell
